# High index of suspicion for brucellosis in a highly cosmopolitan city in southern China

**DOI:** 10.1186/s12879-019-4748-y

**Published:** 2020-01-08

**Authors:** Hai-Yan Ye, Fan-Fan Xing, Jin Yang, Simon Kam-Fai Lo, Ricky Wing-Tong Lau, Jonathan Hon-Kwan Chen, Kelvin Hei-Yeung Chiu, Kwok-Yung Yuen

**Affiliations:** 1 Department of Clinical Microbiology and Infection Control, The University of Hong Kong – Shenzhen Hospital, Shenzhen, China; 2 Department of Microbiology, Queen Mary Hospital, Hong Kong, China; 3 State Key Laboratory of Emerging Infectious Diseases, The University of Hong Kong, Hong Kong, China; 4 Carol Yu Centre for Infection, Li Ka Shing Faculty of Medicine, The University of Hong Kong, Queen Mary Hospital, Hong Kong, China; 5 Department of Microbiology, Li Ka Shing Faculty of Medicine, The University of Hong Kong, Hong Kong, China; 6 The Collaborative Innovation Center for Diagnosis and Treatment of Infectious Diseases, The University of Hong Kong, Hong Kong, China

**Keywords:** Brucellosis, China, Placenta consumption, MALDI-TOF MS, Complication

## Abstract

**Background:**

Brucellosis is one of the most widespread zoonosis in the world. In China, 90% of human brucellosis occurs in six northern agricultural provinces. However, there is a recent increase in the trend of human brucellosis in southern provinces with limited cases reported in the literature. Our study aims to describe the clinical features and epidemiology of brucellosis in a tertiary hospital in southern China.

**Methods:**

A retrospective case series of brucellosis was conducted between January 1, 2014 and October 31. 2018. Cases were identified based on positive Brucella serology by tube agglutination test, or positive culture from clinical specimen identified by Vitek 2 and MALDL-TOF MS. Clinical details of brucellosis including patients’ occupation, risk factors, and complications were analyzed. Clinical characteristics between patients from Guangdong and other provinces were also compared.

**Results:**

A total of 13 cases of laboratory-confirmed brucellosis were identified. 7 (53.8%) of the patients were male, 6 (46.2%) were female, with age ranging from 29 to 73 years old (median age: 51 years). 5 patients (38.5%) were from Guangdong province, while the remaining patients (61.5%) were from other provinces. The commonest risk factors of acquisition were consumption of undercooked meat and goat placenta. Patients from Guangdong province were found to be more likely to have prior placenta consumption. The commonest clinical presentations were fever, osteoarticular pain, urinary symptoms, splenomegaly, and lymphadenopathy. Spondylodiscitis/ peripheral joint arthritis (5 patients, 38.5%) was the most prevalent complication, while extra-osteoarticular complications including abdominal aortitis, hepatosplenic abscess, chest wall abscess, and epididymo-orchitis were observed in 4 other patients. Furthermore, it was demonstrated that MALDI-TOF MS is reliable in *Brucella* identification after additional of reference spectra with standard *Brucella* strain.

**Conclusions:**

Brucellosis, previously thought to be only found in northern China, is now increasingly seen in highly cosmopolitan part of southern China. MALDI-TOF MS in hospitals in China should include reference spectra with standard *Brucella* strain to aid bacterial identification in routine clinical practice. In addition to tuberculosis, typhoid fever and typhus, brucellosis should be considered in patients with fever of unknown origin in this locality.

## Background

Brucellosis is a disease entity caused by a Gram negative coccobacilli of the genus *Brucella*, with *Brucella melitensis* being the commonest implicated agent. Other *Brucella* species including *B. canis*, *B. abortus*, and *B. suis* have also been associated with human disease. Due to the indolent nature of the disease, together with the wide range of animals (such as sheep, cattle, goats, pigs, etc.) being affected by brucellosis, it is one of the most widespread zoonosis in the world [1]. Possible routes of acquisition of brucellosis include consumption of derived food products such as unpasteurized milk and cheese, contact with infectious secretions from animals, and rarely human to human transmission through blood transfusion, sexual contact and organ transplantation [2, 3].

In China, 90% of brucellosis occurs in six northern agricultural provinces including Inner Mongolia, Shanxi, Heilongjiang, Hebei, Jilin, and Shaanxi. However, it is observed that there is a change in the epidemiology of brucellosis in China. Besides the above endemic areas, there is recently an increase in trend of human brucellosis in southern provinces, such as Henan, Guangdong, and Fujian [4]. Retrospective studies in northern China were commonly reported [5, 6], yet similar studies were limited in southern China [7, 8]. This retrospective study aims to include a case series of brucellosis in Shenzhen, a Southern Chinese cosmopolitan city with over 20 million population including a large immigrant population from other parts of China, and to describe the clinical features and epidemiology of this disease in Shenzhen.

## Methods

This is a retrospective study conducted between January 1, 2014 and October 31, 2018 in The University of Hong Kong-Shenzhen Hospital. This 2000-bed multi-specialty hospital was established in 2012 and it provides primary to tertiary medical services to residents of Shenzhen city in both inpatient and outpatient settings.

Diagnosis of brucellosis was suspected through the presence of compatible clinical presentation and investigation findings, and was further confirmed by a positive serology through tube agglutination test or isolation of *Brucella* species from clinical specimens. Serology was performed by Shenzhen Center for Disease Control and Prevention by tube agglutination test using *B. abortus* bacterial suspension. Serum samples were collected and two-fold dilutions were performed using 0.5% phenol saline as diluent, then bacterial antigen suspension was added to the test tube and incubated for 37 °C in a water bath for 20–22 h. A titer of 1:100 is suggestive of acute infection, while a titer of 1:50 is suggestive of chronic infection. The Bac-Tac™ Blood culture system (BacT/ALERT 3D (240), Biomerieux) was used for isolation of *Brucella* species from blood culture and joint aspirate. A Vitek 2 compact 60 system (Biomerieux) was used for bacterial identification in The University of Hong Kong-Shenzhen hospital and Matrix-assisted laser desorption/ionization Time-of-flight mass spectrometry (MALDI-TOF MS) (MicroflexLT/SH, Bruker Daltonics) was used for bacterial identification in Queen Mary Hospital. The reference mass spectra of *B. melitensis* was created by using the ethanol formic acid extraction method in a Biosafety Class 2 cabinet [9].

Clinical details of 13 cases of brucellosis including patients’ occupation, risk factors of acquisition and complications were analyzed and reviewed. Further statistical analysis was performed to compare the differences in clinical characteristics between patients from Guangdong province and other provinces. Statistical analysis was performed using SPSS 24.0. Categorical variables were compared using Chi-square test, continuous variables were analyzed by Mann Whitney U Test, and a *p*-value of less than 0.05 was considered statistically significant.

## Results

A total of 13 cases of brucellosis were identified in the above 58-month study period. The clinical details of the patients were summarized in Table 1. Out of the 13 patients, 7 (53.8%) of the patients were male, 6 (46.2%) were female, with age ranging from 29 to 73 years old (median age: 51 years). 5 patients (38.5%) came from Guangdong province, and 8 patients (61.5%) came from other provinces including Heilongjiang, Inner Mongolia, Fujian, Henan, and Shanxi. The commonest risk factors of acquisition were from consumption of goat placenta (4 patients, 30.8%) and undercooked beef or goat (4 patients, 30.8%). Other risk factors included handling of animal meat (3 patients, 21.3%) and direct goat contact (3 patients, 21.3%). However, no definite risk factors were identified in the remaining two patients (15.4%). Further statistical analysis revealed that patients from Guangdong province were more likely to have consumption of goat placenta (80.0% vs 0.0%, *p* = 0.002) when compared with patients from other provinces (Table 2).

**Table 1:** Summary of the clinical features of the 13 patients with brucellosis

Case	Sex/Age	Occupation	Home town	Risk factor(s)	Presentation	Clinical findings	Complication	Clinical specimen with positive culture	Titer of Brucella Antibody	Treatment
1	F/29y	Clerk	Henan	Contacted goat	Left hip pain for one month	Osteoarticular involvement	Arthritis	Joint fluid	Nil	Doxycycline× 3 months+Rifampin× 3 months+Gentamicin× 14 days
2	M/64y	Retired farmer	Heilongjiang	Contacted goat	Fever, night sweat and urinary urgency for one month	Fever, splenomegaly	Nil	Blood	1:100	Doxycycline×6 weeks+Gentamicin× 7 days
3	M/29y	Businessman	Guangdong	Consumed goat placenta	Fever for 20 days and testicular pain for one week	Fever	Epididymo-orchitis	Blood	Nil	Doxycycline×3 months+gentamiicin× 14 days+Rifampin× 3 months+Ceftriaxone× 14 days
4	M/59y	Unemployed	Heilongjiang	Nil	Fever, abdominal pain and urinary symptoms for one week	Fever, lymphadenopathy, splenomegaly	Intra-abdominal abscess	Blood	1:200	Doxycycline×2 days+Gentamicin×2 days
5	M/64y	Retired worker	Guangdong	Handled and consumed goat meat	Fever with chills and urinary symptoms for one month	Fever, lymphadenopathy	Spondylitis	Blood	1:400	Doxycycline×3 months+Rifampin× 3 months+Gentamicin×7 days
6	M/30y	Office worker	Inner Mongolia	Nil	Recurrent fever for 50 days	Fever, splenomegaly	Nil	Blood	Negative	Doxycycline×6 weeks+Gentamicin×7 days
7	F/51y	Retired farmer	Guangdong	Consumed goat placenta	Fever, severe lower back pain for half a year	Fever, splenomegaly	Nil	Blood	1:400	Doxycycline×6 weeks+Gentamicin×7 days
8	F/31y	Cosmetologist	Fujian	Consumed undercooked beef	Fever with low back pain for one month	Fever, splenomegaly	Spondylitis	Blood	1:100	Doxycycline×4 months+Rifampin×4 months+Gentamicin×14 days
9	F/44y	Pork peddler	Guangdong	Handled and consumed goat placenta	Recurrent lower grade fever for three months	Fever, lymphadenopathy, splenomegaly	Liver and splenic abscess	Blood	1:100	Doxycycline×3 months+Rifampin×3 months+Gentamicin×14 days
10	M/63y	Farmer	Shanxi	Raised goat	Fever and back pain for 10 days	Fever, lymphadenopathy, splenomegaly	Spondylitis	Blood	1:200	Doxycycline×3 months+Rifampin× 3 months+Gentamicin×14 days+Ceftriaxone× 9 days
11	F/33y	Bank clerk	Shanxi	Consumed undercooked beef	Recurrent fever for 17 days	Fever	Nil	Nil	1:100	Doxycycline×6 weeks+Rifampin×6 weeks
12	F/73y	Housewife	Guangdong	Handled and consumed goat placenta	Abdominal pain, low grade fever, dysuria, constipation, low back pain and weight loss one month	Fever, lymphadenopathy, splenomegaly	Possible abdominal aortitis	Blood	1:100	Doxycycline for long-term+Gentamiicin×14 days+Rifampin×6 weeks+Ceftriaxone× 6 weeks
13.	M/56y	Salesman	Henan	Consumed undercooked beef	Recurrent fever with low back pain and chest wall pain	Fever, lymphadenopathy, splenomegaly	Spondylitis, chest wall abscess, osteomyelitis	Blood	1:200	Doxycycline×3 months+Rifampin× 3 months+Gentamicin× 14 days

**Table 2:** Comparison of clinical characteristics between patients from Guangdong province and other provinces

Clinical characteristics	Patient from Guangdong province (%)	Patient from other provinces (%)	*p* value
Demographics			
Sex (Male)	2 (40.0)	5 (62.5)	0.429
Age (Range/ Median)	29–73 (51)	29–64 (44.5)	0.622
Prior exposure/ Risk factors			
Contact with live animals	0 (0.0)	3 (37.5)	0.118
Consumption of animal meat	1 (20.0)	3 (37.5)	0.506
Consumption of goat placenta	4 (80.0)	0 (0.0)	0.002
Symptoms and signs			
Fever	5 (100.0)	7 (87.5)	0.411
Abdominal pain	1 (20.0)	1 (12.5)	0.715
Urinary symptoms	2 (40.0)	2 (25.0)	0.569
Joint pain	0 (0.0)	1 (12.5)	0.411
Low back pain	2 (40.0)	3 (37.5)	0.928
Splenomegaly	3 (60.0)	6 (75.0)	0.569
Lymphadenopathy	3 (60.0)	3 (37.5)	0.429
Complications of infection			
Spondylitis/ Arthritis	1 (20.0)	4 (50.0)	0.279
Epididymo-orchitis	1 (20.0)	0 (0.0)	0.188
Intra-abdominal abscess	1 (20.0)	1 (12.5)	0.715
Abdominal aortitis	1 (20.0)	0 (0.0)	0.188
Investigation results			
Positive culture from clinical specimen	5 (100.0)	7 (87.5)	0.411
Total	5 (38.5)	8 (61.5)	

Concerning the clinical presentation, the most commonly presenting symptoms included fever (92.3%), osteoarticular pain (46.2%) and urinary tract symptoms (30.8%). Other common physical signs in our cohort included splenomegaly (69.2%) and lymphadenopathy (46.2%). The commonest complication in our cohort was spondylodiscitis/ peripheral joint arthritis (5 patients, 38.5%), while extra-osteoarticular complications including abdominal aortitis, hepatosplenic abscess, chest wall abscess, and epididymo-orchitis were observed in 4 other patients, with the remaining 4 patients with no definite focal involvement. No statistically significant differences were observed in terms of clinical presentation and complications when compared between patients from Guangdong provinces and other provinces (Table 2).

**Fig. 1:**
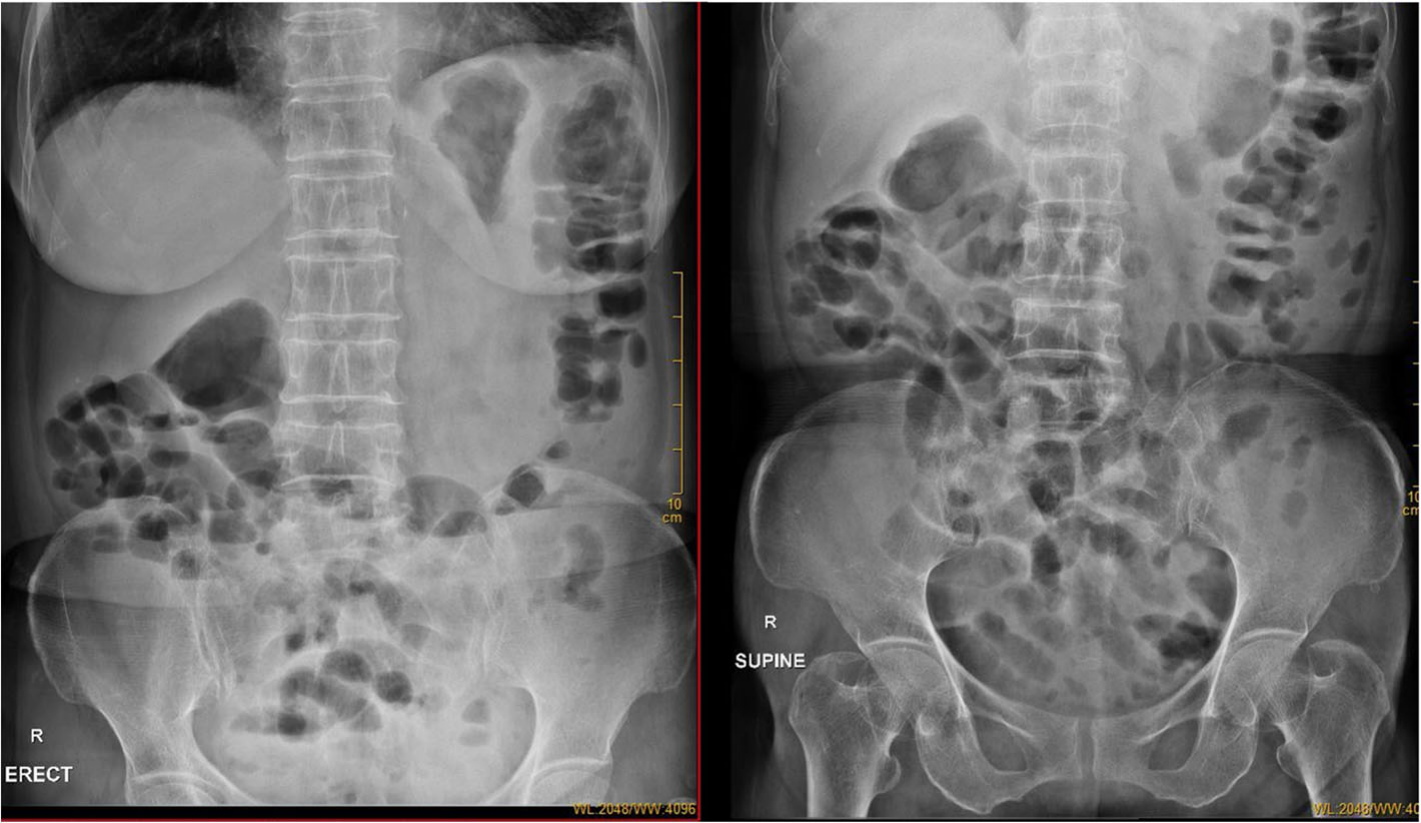
Abdominal X-ray in supine and erect position. Prominent bowel loops were noted in the Abdominal X-ray, together with multiple fluid levels in erect Abdominal X-ray suggestive of underlying ileus. Psoas shadow was obscured suggestive of possible retroperitoneal pathology, such as infected abdominal aortic aneurysm

**Fig. 2:**
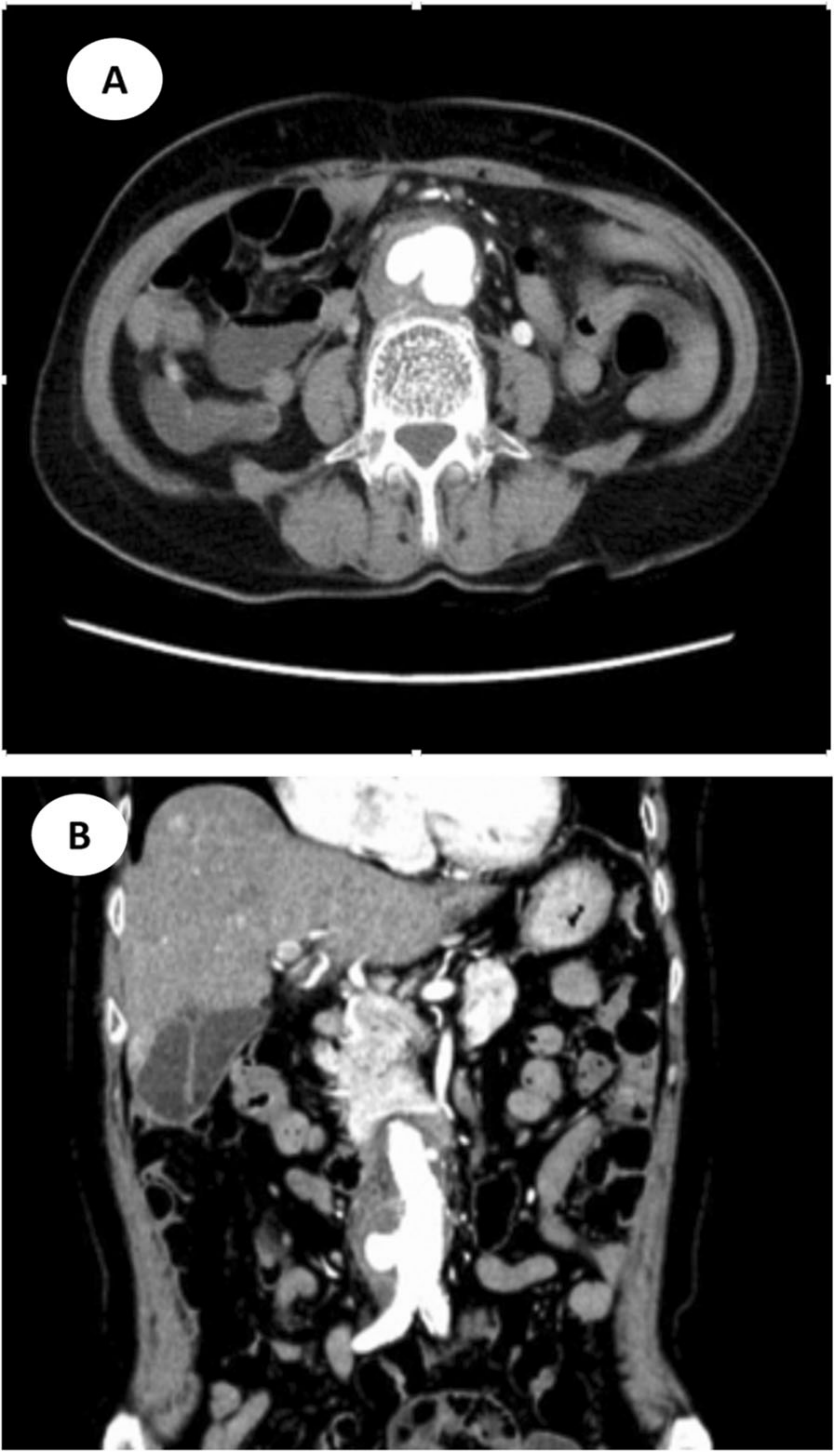
Computed tomographic angiography (CTA) of abdomen before surgery (**a**) Axial view (**b**) Coronal view. CTA during contrast phase showed abdominal aortic aneurysm with atherosclerotic changes, together with mural thrombus and penetrating ulcer

**Fig. 3:**
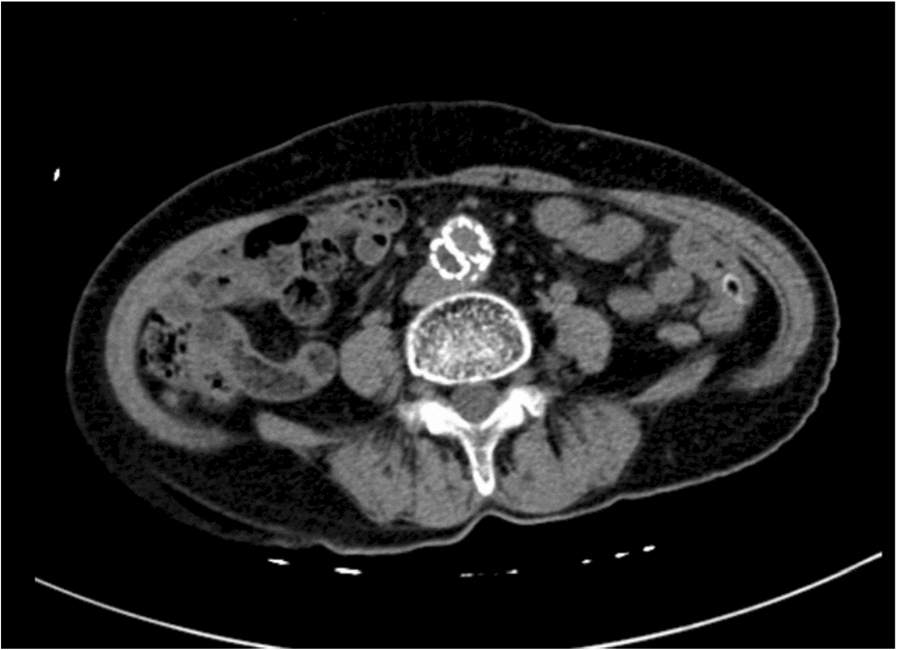
Computed tomographic angiography (CTA) of abdomen after surgery. Emergency endovascular repair was performed with a bifurcated stent-graft inserted as shown in the CTA

Among all of the above complications, the most life-threating complication was abdominal aortitis, as demonstrated by the patient labelled case 12. This patient is an elderly woman, with history of hypertension and cerebral infarction on long term aspirin (acetylsalicylic acid), losartan, metoprolol, and betahistine. She had history of handling and consumption of goat placenta on multiple occasions during the past one year. She first presented to our hospital with constitutional symptoms (fatigue for 6 months, weight loss of 5 kg in one month and fever), right-sided abdominal pain radiating to the back for 1 week and non-specific symptoms such as cough, dysuria, and constipation. A supine abdominal X-ray revealed multiple air-fluid levels in the small bowel (Fig. 1). Computed tomographic angiography (CTA) showed an abdominal infrarenal aortic aneurysm with penetrating ulcer, together with pseudoaneurysm, and presence of lymphadenopathy (Fig. 2a and b), which were not known prior to this admission. Emergency endovascular repair was successfully performed with a bifurcated stent-graft inserted (Fig. 3). Although the above development of aortic aneurysm could be accounted by underlying vascular risk factors, with the development of abdominal symptoms only 1 week prior to admission, presence of lymphadenopathy in CT scan, together with *B. melitensis* isolated from blood culture, overall clinical picture was suggestive of possible abdominal aortitis secondary to *B. melitensis*. Therefore, she was given intravenous antibiotics for 6 weeks, then antibiotics were subsequently stepped down to oral doxycycline for long term suppressive therapy. Currently the patient is still under follow up in our clinic for one year, with good clinical outcome and no recurrence.

**Fig. 4:**
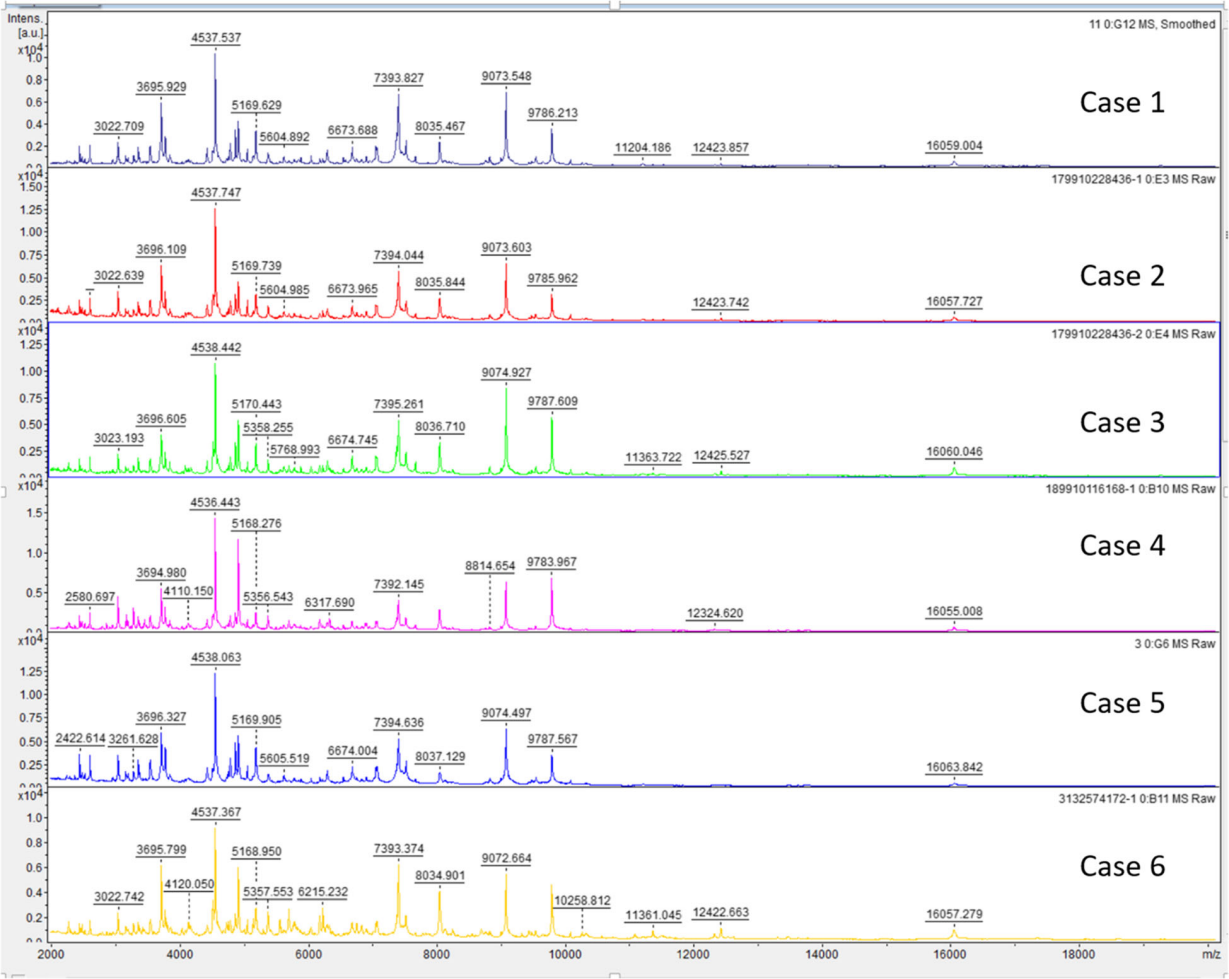
MALDI-TOF MS spectra of the first 6 cases in the range from 2000 to 20,000 Da. Bacterial colonies from clinical specimen of the Cases 1–6 were identified by MALDI-TOF MS as *Brucella melitensis*, with common specific peaks with reference spectra for all the strains at 3023 Da, 3696 Da, 4537 Da, 5170 Da, 6674 Da, 7394 Da, 8037 Da, 9074 Da, 9786 Da, 12,425 Da and 16,060 Da

**Fig. 5:**
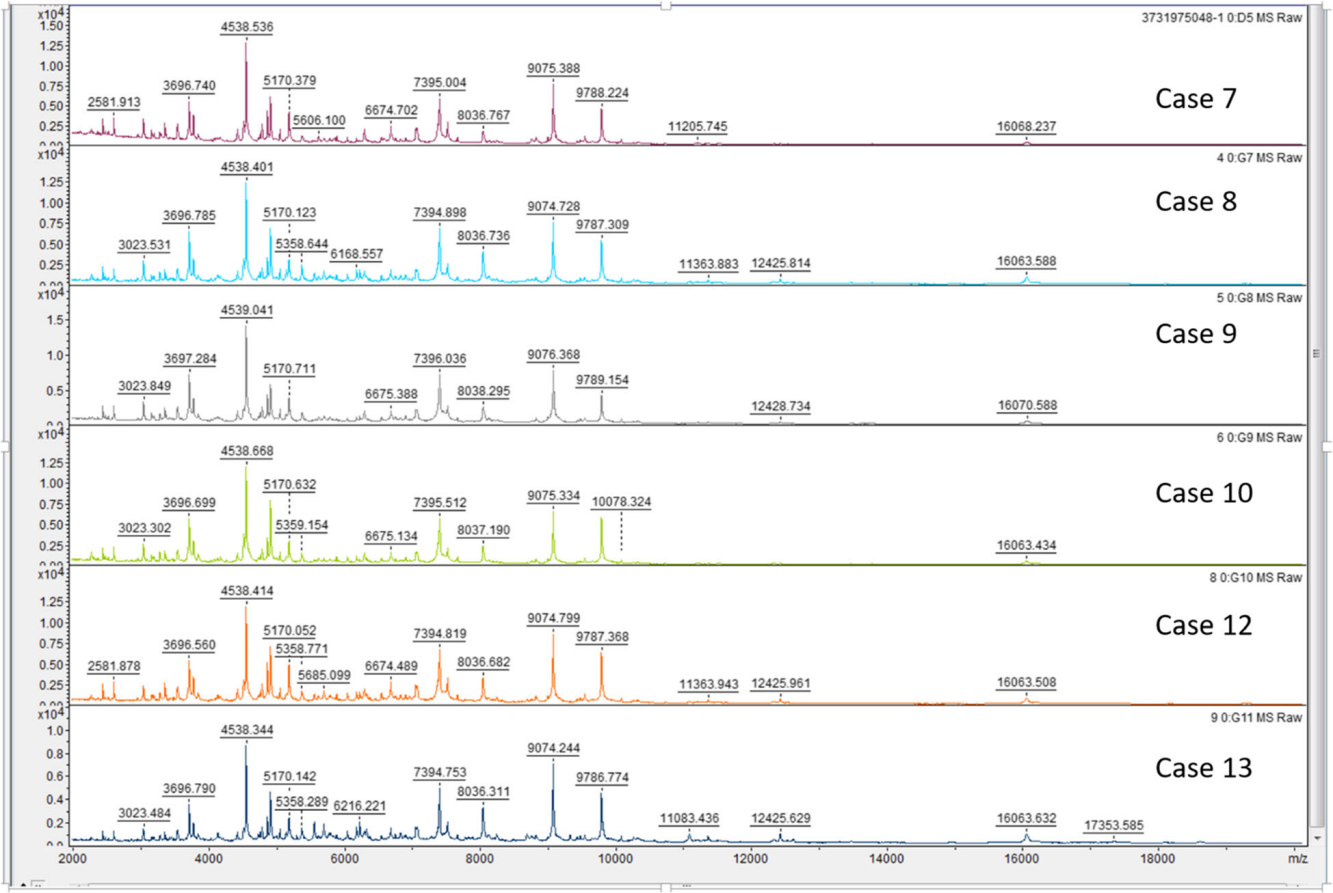
MALDI-TOF MS spectra of the last 6 cases in the range from 2000 to 20,000 Da. Bacterial colonies from clinical specimen of the Cases 7–10, 12–13 were identified by MALDI-TOF MS as *Brucella melitensis*, with common specific peaks with reference spectra for all the strains at 3023 Da, 3696 Da, 4537 Da, 5170 Da, 6674 Da, 7394 Da, 8037 Da, 9074 Da, 9786 Da, 12,425 Da and 16,060 Da

*Brucella* species was isolated from the blood culture from eleven patients and joint fluid culture from one patient. All 12 strains were identified by MALDI-TOF MS, and visual inspection of these spectra from all the strains revealed a high similarity with reference spectra of *B. melitensis* (Fig. 4-5), with common specific peaks for all the strains at 3023 Da, 3696 Da, 4537 Da, 5170 Da, 6674 Da, 7394 Da, 8037 Da, 9074 Da, 9786 Da, 12,425 Da and 16,060 Da. *B. melitensis* with confidence scores above 2.0 were obtained for all isolates, and if other bacteria such as *Ochrobactrum*, *Pseudomonas* and *Enterobacteriaceae* were included in the final MALDI-TOF MS results table, the confidence scores were less than 1.5. In addition, all strains were further identified by Vitek 2 compact system, with the result compatible with *B. melitensis.*

No positive culture from clinical specimen could be obtained from 1 of the 13 patients, while her Brucella antibody titer was found to be high with her clinical disease responded to doxycycline. Among the remaining 11 patients with serology performed by standard tube agglutination test, 10 patients (90.9%) showed a positive titer with the remaining patient (9.1%) having a negative titer despite a positive blood culture.

These patients were treated with various combinations of antibiotics depending on the organ of involvement, side effects, and tolerability of antibiotics. The shortest duration of antibiotics was 6 weeks for patients with uncomplicated disease, and 3–4 months for osteoarticular involvement, abscess and epididymo-orchitis; with the exception of case 4 who ceased treatment after 2 days of antibiotics. The patient with intravascular infection such as abdominal aortitis was put on long term oral suppressive therapy after endovascular surgery. There were 3 (23.1%) patients with uncomplicated disease receiving doxycycline plus gentamicin, whereas 1 (7.7%) received doxycycline plus rifampin; 5 (38.5%) patients received a combination of doxycycline, gentamicin plus rifampin, and 3 (23.1%) patients with more complicated disease received a combination of ceftriaxone and doxycycline, gentamicin plus rifampin.

## Discussion

Guangdong province is one of the areas in China with the lowest incidence of brucellosis in the past [10]. However, it was observed by different studies that there is an emergence of cases of brucellosis in southern China [11]. Shenzhen, a special economic zone in China locating in the Guangdong province, has an estimated population of 12 million people, including a large migrant population from other regions of China. In our study, 8 out of 13 patients (61.5%) came from other provinces including Heilongjiang, Inner Mongolia, Fujian, Henan, Shanxi. Among the five provinces, three are high-risk regions of human brucellosis in China, with three patients having history of goat contact at their hometown. The above fact illustrated that the movement of people from northern to southern China may explain the current change in the epidemiology of brucellosis in China.

Furthermore, live animals and raw meat products are frequently transported from northern to southern China, these actions put ordinarily low-risk population at much higher risk when they consume or handle infected animal products. In our study, 4 out of 13 (30.8%) patients had history of consumption of undercooked meat. The chance of exposure to undercooked meat could be related to change in eating habit, such as having hotpot or western cuisine that sometimes food could be served raw. The risk could be further increased if there is a lack of means of livestock quarantine, therefore leading to infected animal products readily crossing the border between provinces [4].

In addition to that, 4 out of 13 (30.8%) patients had history of consumption of goat placenta. According to traditional Chinese medicine theory, consumption of placenta is believed to be able to boost immunity and nutrition, therefore leading to an increase risk of acquiring brucellosis in the Chinese population. We also showed in our cohort that patients from Guangdong province had statistically significant prior exposure to consumption of goat placenta when compared with patients from other provinces. The above findings were compatible with the previous survey in Guangdong province that more than 20% of cases of brucellosis had history of goat placenta consumption [4]. The above difference in the exposure between different provinces could be due to the special eating habits in southern part of China, which were also being observed in other studies [8, 11, 12]. Furthermore, previous animal models have shown that the bacterial load of *Brucella* species is extremely high in genital organs during parturition. From a pregnant mouse model for vertical transmission of *B. melitensis*, it was shown that a higher degree of bacterial colonization was found in the placenta than in the spleen and fetus [13]. During acute brucellosis in pregnant cows, up to 85% of the bacteria are found in cotyledons, placental membranes, and allantoic fluid [14]. Thus, placenta contact possesses a greater risk to patients in acquiring brucellosis. With the above consideration of risk factors, human brucellosis would be expected to increase in southern China in the future.

Osteoarticular involvement is the commonest complication observed similar to other studies [15, 16], with 1 patient developed arthritis and 4 patients developed spondylodiscitis. Cardiovascular involvement is uncommon [17, 18], quoted to be < 2% of in most studies, and it usually manifests as culture negative endocarditis. Although cardiovascular involvement is rare, it remains the principal cause of mortality, usually involving the aorta causing aortitis. Arteriosclerotic plaques serve as the site of attachment for microorganisms due to their irregular surfaces, therefore artherosclerosis has been considered to be a risk factor of mycotic aneurysm and aortitis. From the CTA imaging of case 12, certain amount of arteriosclerotic plaques could be observed in the aorta. Considered our study had a case of possible abdominal aortitis (7.7%) in the hypoendemic regions, it is likely that numerous cases went undiagnosed in the hyperendemic regions, thus underestimating the true incidence of the disease. Due to the lack of awareness of brucellosis as the etiology of ruptured aneurysm, further microbiological testing would be required for intraoperative specimen if available.

MALDI-TOF MS has been suggested to be a fast and reliable method for bacterial identification [19]. Databases have been developed to include the main pathogenic microorganisms such as the In Vitro Diagnostic Database adopted by BioMurex, thus allowing the use of this method in routine bacterial identification in daily clinical practice. However, *Brucella* species have not yet been incorporated to some of the main available database, especially in hypoendemic regions where isolation of Brucella is infrequent, therefore leading to the limitation of misidentification of Brucella species as *Ochrobactrum* species in these clinical settings. In our study, the 12 Brucella isolates were subjected to direct comparison using Vitek 2 compact system and MALDI-TOF MS, with both systems showing the same result of *B. melitensis*. This not only conformed to previous literature that *B. melitensis* is the commonest species causing human brucellosis [20], but also suggested that MALDI-TOF MS is reliable and rapid in Brucella identification after additional of reference spectra with standard *Brucella* strain.

One of the limitations of our study is the difficulty in determination of the definitive route of transmission in all of the cases. As this study is retrospective in nature, we were unable to obtain environmental or food samples for isolation of *Brucella* species in these specimens, and to further perform phylogenetic analysis to confirm the definitive link of transmission, therefore only risk factors of acquisition were analyzed.

## Conclusion

In summary, due to the increasing number of migrant populations from northern to southern China, changes in lifestyle and habit of consumption of goat placenta for traditional Chinese medicine use, the incidence of brucellosis is expected to increase in the future in southern China. MALDI-TOF MS in hospitals in China should include reference spectra with standard *Brucella* strain to aid bacterial identification in routine clinical practice. In addition to tuberculosis, typhoid fever and typhus, *Brucella* infection should be considered as a differential diagnosis for patients with pyrexia of unknown origin in southern China. Cardiovascular involvement due to brucellosis maybe previously considered as uncommon, however, with the aging population and growing population of patients with underlying cardiovascular disease, the possibility of cardiovascular involvement should not be overlooked.

## Data Availability

The datasets used and analyzed during the current study are available from the corresponding author on reasonable request.

## Abbreviations

CTA: Computed tomographic angiography
Da: Dalton
MALDI-TOF MS: Matrix-assisted laser desorption/ionization Time-of-flight mass spectrometry

## References

[1] Bosilkovski M, Dimzova M, Grozdanovski K (2009). Natural history of brucellosis in an endemic region in different time periods. Acta Clin Croat.

[2] Wang W, Liao Q, Wu X, Hou S, Wang Y, Wu J (2015). Potential risk of blood transfusion-transmitted brucellosis in an endemic area of China. Transfusion.

[3] Tuon FF, Gondolfo RB, Cerchiari N (2017). Human-to-human transmission of Brucella - a systematic review. Tropical Med Int Health.

[4] Zhong Z, Yu S, Wang X, Dong S, Xu J, Wang Y (2013). Human brucellosis in the People's Republic of China during 2005–2010. Int J Infect Dis.

[5] Zhang X, Wang Z, Mu G, Wang T (2015). Brucellosis control in Northeast China: a long way to go. Public Health.

[6] Zhang WY, Guo WD, Sun SH, Jiang JF, Sun HL, Li SL (2010). Human brucellosis, Inner Mongolia. China Emerg Infect Dis.

[7] Wong TM, Lou N, Jin W, Leung F, Leung F, To M (2014). Septic arthritis caused by Brucella melitensis in urban Shenzhen. China: a case report J Med Case Rep.

[8] Chen S, Zhang H, Liu X, Wang W, Hou S, Li T (2014). Increasing threat of brucellosis to low-risk persons in urban settings. China. Emerg Infect Dis.

[9] Freiwald A, Sauer S (2009). Phylogenetic classification and identification of bacteria by mass spectrometry. Nat Protoc.

[10] Kong W (2018). Brucellosis infection increasing in southern China. Eur J Intern Med.

[11] Chen JD, Ke CW, Deng X, Jiang S, Liang W, Ke BX (2013). Brucellosis in Guangdong Province, People's Republic of China, 2005-2010. Emerg Infect Dis.

[12] Guo J, Lai W, Wu Y, Mu X (2018). Eating goat’s placenta and brucellosis caused by Brucella melitensis. Int J Clin Exp Med.

[13] Wang Z, Wang SS, Wang GL, Wu TL, Lv YL, Wu QM (2014). A pregnant mouse model for the vertical transmission of Brucella melitensis. Vet J.

[14] Anderson TD, Meador VP, Cheville NF (1986). Pathogenesis of Placentitis in the goat inoculated with Brucella abortus. I. Gross and histologic lesions. Vet Pathol.

[15] Buzgan T, Karahocagi MK, Irmak H, Baran AI, Karsen H, Evirgen O (2010). Clinical manifestations and complications in 1028 cases of brucellosis: a retrospective evaluation and review of the literature. Int J Infect Dis.

[16] Kose S, Serin Senger S, Akkoclu G, Kuzucu L, Ulu Y, Ersan G (2014). Clinical manifestations, complications, and treatment of brucellosis: evaluation of 72 cases. Turk J Med Sci.

[17] Chen SL, Kuo IJ, Fujitani RM, Kabutey NK (2017). Endovascular Management of Concomitant Thoracic and Abdominal Aortic Ruptures Resulting from Brucellosis Aortitis. Ann Vasc Surg.

[18] Cascio A, De Caridi G (2012). Lentini, Benedetto F, Stilo F, Passari G, Iaria C, et al. involvement of the aorta in brucellosis: the forgotten, life-threatening complication. A systematic review. Vector Borne Zoonotic Dis.

[19] Ferreira L, Vega S, Sanchez-Juanes F, Gonzalez M, Herrero A, Muniz MC (2010). Identifying bacteria using a matrix-assisted laser desorption ionization time-of-flight (MALDI-TOF) mass spectrometer. Comparison with routine methods used in clinical microbiology laboratories. Enferm Infecc Microbiol Clin.

[20] Pappas G, Panagopoulou P, Christou L, Akritidis N (2006). Brucella as a biological weapon. Cell Mol Life Sci.

